# Endogenous Nitric Oxide-Releasing Microgel Coating Prevents Clot Formation on Oxygenator Fibers Exposed to In Vitro Blood Flow

**DOI:** 10.3390/membranes12010073

**Published:** 2022-01-06

**Authors:** Patrick Winnersbach, Aisa Hosseinnejad, Thomas Breuer, Tamara Fechter, Felix Jakob, Ulrich Schwaneberg, Rolf Rossaint, Christian Bleilevens, Smriti Singh

**Affiliations:** 1 Department of Anesthesiology, University Hospital RWTH Aachen, Pauwelsstraße 30, 52074 Aachen, Germany; pwinnersbach@ukaachen.de (P.W.); tfechter@ukaachen.de (T.F.); rrossaint@ukaachen.de (R.R.); 2 DWI—Leibniz-Institute for Interactive Materials e.V., Forckenbeckstr. 50, 52056 Aachen, Germany; hosseinnejad@dwi.rwth-aachen.de (A.H.); jakob@dwi.rwth-aachen.de (F.J.); u.schwaneberg@biotec.rwth-aachen.de (U.S.); 3 Department of Intensive Care and Intermediate Care, University Hospital RWTH Aachen, Pauwelsstraße 30, 52074 Aachen, Germany; tbreuer@ukaachen.de; 4 Institute for Biotechnology, RWTH Aachen University, Worringerweg 3, 52074 Aachen, Germany; 5 Max-Planck-Institut für medizinische Forschung, Jahnstraße 29, 69120 Heidelberg, Germany

**Keywords:** blood-contacting surfaces, ARDS, PMP fibers, microgel coating, platelet activation, anti-thrombogenicity, systemic anticoagulation

## Abstract

Background: Clot formation on foreign surfaces of extracorporeal membrane oxygenation systems is a frequent event. Herein, we show an approach that mimics the enzymatic process of endogenous nitric oxide (NO) release on the oxygenator membrane via a biomimetic, non-fouling microgel coating to spatiotemporally inhibit the platelet (PLT) activation and improve antithrombotic properties. This study aims to evaluate the potential of this biomimetic coating towards NO-mediated PLT inhibition and thereby the reduction of clot formation under flow conditions. Methods: Microgel-coated (NOrel) or bare (Control) poly(4-methyl pentene) (PMP) fibers were inserted into a test channel and exposed to a short-term continuous flow of human blood. The analysis included high-resolution PLT count, pooled PLT activation via β-Thromboglobulin (β-TG) and the visualization of remnants and clots on the fibers using scanning electron microscopy (SEM). Results: In the Control group, PLT count was significantly decreased, and β-TG concentration was significantly elevated in comparison to the NOrel group. Macroscopic and microscopic visualization showed dense layers of stable clots on the bare PMP fibers, in contrast to minimal deposition of fibrin networks on the coated fibers. Conclusion: Endogenously NO-releasing microgel coating inhibits the PLT activation and reduces the clot formation on PMP fibers under dynamic flow.

## 1. Introduction

Extracorporeal membrane oxygenation (ECMO) is the last therapeutic option for patients with isolated or combined acute cardiopulmonary failure [1]. Venoarterial ECMO (VA-ECMO) primarily provides circulatory support in cardiogenic shock [2], while venovenous ECMO (VV-ECMO) is mainly used in patients suffering from isolated pulmonary conditions, such as acute respiratory distress syndrome (ARDS) [3].

The main purpose of VV-ECMO therapy is the oxy- and decarboxygenation of blood. Therefore, the patients’ blood circulation needs to be connected to extracorporeal circulation (ECC) and pass an oxygenator for gas exchange [4,5].

The oxygenator, mainly consisting of hollow fibers, which represent the ECCs largest blood-contacting foreign surface, is the core component of an ECMO system. Hollow fibers are commonly made of hydrophobic polymers, including poly(4-methyl-1-pentene) (PMP) commercially known as TPX [6]. However, the nature of these materials usually gives rise to life-threatening complications in response to the foreign body-blood interaction. The adverse interaction with blood leading to the formation of thrombi is initiated by a coagulation cascade set in by protein adsorption on the contact surface [4,5,7].

To prevent clot formation on parts of the ECMO system, especially in the oxygenator, systemic anticoagulation, usually achieved by sufficient administration of intravenous heparin, is indispensable [8] but is also a cause of bleeding complications [9,10,11]. Even though systemic anticoagulation with heparin has indirect effects on PLTs, such as the inhibition of PLT activation via inactivation of thrombin [12], it is not sufficient to prevent PLT activation induced by protein adsorption on foreign surfaces [13].

PLT activation is followed by adherence to the surface and amplified coagulation by the release of procoagulant-signaling molecules, such as adenosine diphosphate (ADP) or Serotonin [14,15]. This cascade is concomitantly related to the huge foreign surface and high shear stress within the oxygenator [16,17].

Different strategies of direct inhibition of PLT activation and subsequent pro-coagulatory effects are well known, but due to the aggravation of bleeding issues, effective systemic PLT inhibition is not applicable for ECMO [18,19].

The preeminent solution to provide sufficient PLT mitigation and overcome associated bleeding complications is the application of self-regulated and localized PLT inhibition in the ECC, most importantly on the oxygenator membranes without affecting the PLT concentration downstream.

Nitric oxide (NO), contributing to the thromboresistant properties of the blood vessels endothelium, is an effective inhibitor of PLT activation and adhesion [20]. It is released from the endothelial cells mediated by endothelial NO synthase (eNOS), which increases the intracellular concentration of cyclic guanosine monophosphate (cGMP) in PLTs. This leads to a reduced quantity and affinity of fibrinogen binding sites (GPIIb/IIIa receptor) on PLT membranes [21].

There are many different strategies to locally provide NO for precise PLT inhibition. A simple approach is the delivery of NO via sweep gas [22,23]. The direct generation of NO can also be achieved by surface modification, whereas the NO-donor species (e.g., diazeniumdiolates) are impregnated, blended or covalently incorporated in polymeric scaffolding blood-contacting materials [20,24]. However, the performance of these strategies is still restricted by a variety of limiting factors (e.g., accessible surface area, the lifetime of NO, synthesis complications, toxicity), but above all, they suffer from the inability to counteract the non-specific protein fouling on the surface.

The most recent approach is the enzyme mimetic catalytic generation of NO, which utilizes the release of NO from endogenous S-nitrosothiols (RSNO) [25,26]. As endogenous NO-reservoirs, RSNO proteins provide an ever-present substrate for NO generation in blood, which solves the problem of consumption [25,27,28]. Therefore, a surface coating with an endogenous NO-release functionality seems to be promising for long-term application on oxygenator membranes.

**Scheme 1:**
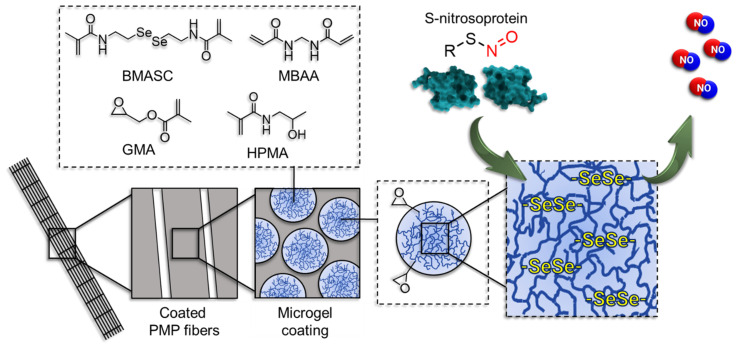
The mechanism of endogenous NO-release mediated by the microgel coating on poly(4-methyl-1-pentene) (PMP) fibers (not to scale). Microgels are prepared using *N*-(2-hydroxypropyl)methacrylamide (HPMA) and glycidyl methacrylate (GMA) via free-radical emulsion polymerization in an inverse miniemulsion with *N*,*N*’-bis(methacryloyl)selenocystamine (BMASC) and *N*,*N*’-methylenebis(acrylamide) (MBAA) as a crosslinker.

An enzyme mimetic non-fouling microgel coating for endogenous NO release was recently developed by this group [29]. The microgels with a highly hydrophilic base are incorporated with diselenide crosslinks [30,31]. While the hydrophilicity provides the protein repellency, the reversible diselenide bridges mimic the enzymatic function of the selenium-bearing glutathion peroxide (GPx) to cleave the S-nitroso bond (S–NO) of S-nitrosoproteins. This results in a continuous rate of NO-release from the in vivo pool of NO in blood. The introduction of epoxy groups to the microgel structure facilitates the successful attachment of microgels to PMP membranes mediated by an anchor-peptide (Scheme 1). Anchor peptides are conjugated via a free and accessible HS-group to the microgels [32,33]. At static conditions, the coating has proven successful in the prevention of protein adsorption, the sustained release of NO and subsequent mitigation of PLT-activation.

To challenge the PLT-inhibitory effect of this microgel coating under a dynamic condition, as found in real ECMO, this in vitro study aims to investigate and analyze this microgel coating under flow conditions, which increases protein deposition and PLT adhesion [34]. For this purpose, systematically characterized microgel-coated PMP fibers were inserted into a test channel and exposed to blood flow (1 mL min^−1^) for a short-term measurement and analyzed macroscopically and microscopically (confocal laser scanning microscopy (CSLM) and scanning electron microscopy (SEM)) to visualize thrombus formation. Additionally, continuous PLT count and pooled PLT activation via β-Thromboglobulin (β-TG) concentration were analyzed to directly evaluate the inhibitory effect of NOrel coating on PLTs in blood flow compared to uncoated fibers taken as a reference.

## 2. Materials and Methods

### 2.1. Microgel Synthesis and Coating

Nitric oxide-releasing microgels (NOrel-MGs) were synthesized using *N*-(2-hydroxypropyl)methacrylamide (HPMA) as a monomer and diselenide-bearing *N*,*N*’-bis(methacryloyl)selenocystamine (BMASC) and *N*,*N*’-methylenebis(acrylamide) (MBAA) as crosslinkers. The synthesis of HPMA, BMASC and NOrel-MGs was reported in our previous work [29]. Briefly, the microgels were fabricated in an inverse miniemulsion through free-radical emulsion polymerization. HPMA was the main constituent of the microgels, doubly crosslinked with BMASC [30,31] and MBAA to improve the preservation of functional microgelic systems. To facilitate the surface functionalization, glycidyl methacrylate (GMA) was introduced to the microgel structure. During the synthesis of the microgels, the ratio of HPMA to GMA was kept in the molar ratio of 38:1.

**Scheme 2:**
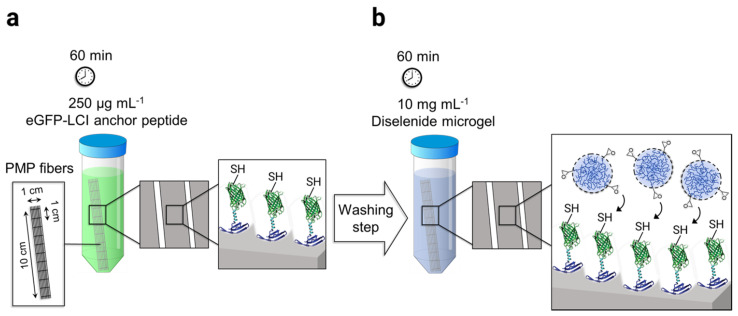
Schematic representation of the microgel-coating procedure on the poly(4-methyl-1-pentene) (PMP) fiber mat: (**a**) Enhanced green fluorescent protein–liquid chromatography peak I (eGFP-LCI) binding to the fibers by immersion of the mat in 250 µg mL^−1^ of the eGFP-LCI followed by a washing step. (**b**) The immobilization of microgels on the eGFP-LCI coated fibers was achieved using the thiol-epoxy click reaction.

The adhesion of microgels on PMP hollow fibers was mediated by the surface-affine anchor peptide eGFP-LCI provided by the Schwaneberg group. The production of recombinant eGFP-LCI using *E. coli* is reported elsewhere in the literature [35]. The process of microgel immobilization was performed in two steps. First, the knitted mat of PMP fibers consisting of 20 threads, 10 cm long, were incubated in 15 mL of 250 µg mL^−1^ eGFP-LCI solution in PBS for 60 min in the dark (Scheme 2a). Subsequently, the fibers were thoroughly washed three times using deionized water. The eGFP-LCI-treated fibers were then incubated in the 10 mg mL^−1^ microgel solution in PBS (pH 7.4) for a further 60 min in the dark (Scheme 2b) followed by the washing step as before. The microgel-coated fibers were dried under a gentle stream of N_2_ and stored at 4 °C for further analysis.

### 2.2. Microgel Coating Characterization on PMP Fibers

#### 2.2.1. Before Contact with Blood Flow

**Figure 1:**
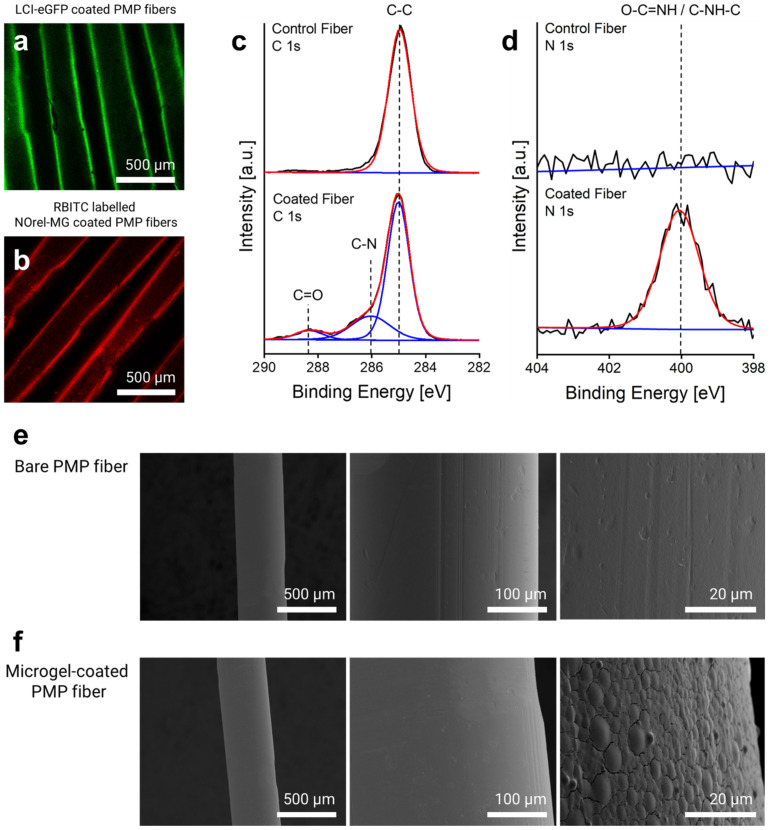
Microgel-coating characterization: CLSM imaging of (**a**) eGFP-LCI-treated and (**b**) Microgel-coated PMP fibers. High-resolution (**c**) C1s and (**d**) N1s X-ray Photoelectron Spectroscopy (XPS) spectra of the control (uncoated) and microgel-coated fibers reveal the corresponding signals. SEM micrographs of the surface morphology of the PMP fiber (**e**) before and (**f**) after microgel coating.

The binding of eGFP-LCI and microgels labeled with rhodamine–B-isothiocyanate (RBITC) on the fibers was assessed by confocal laser scanning microscopy (CLSM) using Leica TCS SP8 CLSM (Leica Microsystems, Wetzlar, Germany). The fluorescence intensity of the enhanced green fluorescent protein (eGFP) (excitation λ = 488, emission λ = 500–550 nm) and the fluorescently labeled microgels (excitation λ = 561, emission λ = 575–610 nm) was measured using an argon laser with 10% intensity and PMT2 detector. A uniform and homogeneous coating layer of eGFP-LCI (Figure 1a), as well as of RBITC-labeled microgels (Figure 1b), can be seen on the fiber surface, respectively, that corroborates the proposed coating process.

The elemental composition of the fibers before and after microgel coating was analyzed by X-ray Photoelectron Spectroscopy (XPS) using Kratos Ultra Axis (Kratos Analytical Ltd., Manchester, UK). For this purpose, 1 × 1 cm^2^ knitted mat of PMP fibers consisting of 20 threads coated with microgels was prepared and fixed on a 1 × 1 cm^2^ silicon wafer. The sample was excited with monochromatic Al-Kα_1,2_ radiation (1486.6 eV), and the resulting spectra were evaluated with CasaXPS processing software Version 2.3.15 (Casa Software Ltd., Devon, UK). The binding energy (BE, eV) was corrected with C 1 s (285.0 eV) as the standard. The changes in the elemental composition of the fibers, particularly in the carbon and nitrogen content before and after microgel coating, revealed the immobilization of the microgels on the surface. As shown in Figure 1c, the new signals (at 286.0 and 288.3 eV) in the C1s spectrum appeared after microgel coating that corresponds to C–N and C=O, respectively, which arise from the amide bond of the microgels. The comparison of N1s spectra before and after coating further indicated the presence of the microgels on the surface (Figure 1d), as only the surface of the coated fiber shows a dominant amide peak (O=C–NH) at 400.1 eV. The calculated atomic content of nitrogen also affirmed that the nitrogen contribution increased up to 7.2% after microgel coating, while the bare fiber lacked any contributions of nitrogen.

Scanning electron microscopy (SEM) was used to ascertain the morphology and distribution of the microgel coating using a Hitachi S-3000 electron microscope (Hitachi, Tokyo, Japan) with an acceleration voltage between 1 and 10 kV. For analysis, the microgel-coated single fibers were horizontally fixed on a cylindrical slot holder with a 5 mm wide slot. The fixed fibers were then sputter-coated with a 10 nm layer of gold/palladium (80:20) using an ACE 600 sputter coater (Leica, Germany). Uncoated bare fibers were used as a control. Figure 1e shows the surface characteristics of the bare PMP fibers with their defined inherent roughness. After microgel coating, the roughness of the fibers decreased, and in the higher magnification, a uniform distribution of the microgels homogeneously covering the surface is discernible (Figure 1f). The flattened shape of the microgels is attributed to their dry state due to the water loss and high affinity for the surface after the LCI treatment and thereby expanding/broadening on the surface [36].

#### 2.2.2. Subsequent Analysis after Contact with Blood Flow

The formation of a clot on the surface of both groups of the fibers (control bare fibers and microgel-coated fibers) after 48 min of contact with blood flow was inspected by SEM (S-3000, Hitachi, Tokyo, Japan) using an acceleration voltage between 1 and 10 kV. Prior to analysis, the samples were fixed with the 4% paraformaldehyde (PFA) solution in PBS for 15 min, followed by a dehydration step. The samples were immersed for 5 min, respectively, in 0%, 30%, 50%, 70% and 100% mixtures of PBS:ethanol and finally dried using hexamethyldisilazane (HMDS). After the fixing step, the fibers were horizontally affixed at a distance from each other on a cylindrical slot (5 mm) holder and sputter-coated by a 10 nm layer of gold/palladium (80:20) using an ACE 600 sputter coater (Leica, Germany).

### 2.3. Protein Fouling on Microgel Coated Fibers

The protein repletion of the microgel coating was evaluated by incubation of a microgel-coated 1 × 1 cm^2^ knitted mat of PMP fibers with human blood serum (HBS), H4522, for 60 min at 37 °C. The membrane was thoroughly washed with deionized water and dried under a mild stream of N_2_, followed by measuring at XPS. The same procedure of protein fouling test and the further XPS analysis was performed on a 1 × 1 cm^2^ bare knitted mat of PMP fibers as a control. This analysis shows the protein fouling/deposition on the surface of bare fibers accompanied by significant changes in the surface composition effected by the nitrogen content, whereas it cannot be seen on the surface of the microgel-coated fibers (Figure S2). This is further corroborated by the atomic content analysis of XPS calculations (Table S1), where the nitrogen contribution in microgel-coated fiber shows negligible changes, while it drastically increased up to 9.7% in control.

Microgels are soft, colloidal particles with an open structure and have a high ability to absorb water, which reduces the interfacial tension with blood [37]. Using microgels as a coating material is a new class of hydrophilic surface coatings that can be used to passively reduce the protein adsorption on the surface. The microgels proposed in this work were synthesized using HPMA. HPMA is a simple hydroxyl methacrylamide known for its high hydrophilicity and excellent antifouling property. Its low surface charge, along with the strong ability to form multiple hydrogen bonds via its hydroxyl and amide monomeric units, leads to a dense hydration top-layer, which enthalpically prevents cell and protein adhesion on the coated surface of polymeric substrates [38,39].

### 2.4. Assessment of NO-Release

To assess and quantify the NO released from the prepared microgel coating, the spectrophotometric Griess test was used. For this, the Griess reagent was made of mixing equal volumes of *N*-(1-naphthyl)ethylenediamine dihydrochloride and sulfanilic acid. A total of 20 knitted microgel-coated PMP fibers of 1 × 1 cm^2^ area were prepared followed by incubation in a mixture of 0.5 mL L-Glutathione (GSH) (1 mM), 0.5 mL S-nitrosoglutathione (GSNO) (7 µM), 0.2 mL Griess reagent and 1.8 mL distilled water. GSH and GSNO used in a co-related bioavailable concentration [40,41] are the physiological model supplements needed for triggering the catalytic function of the microgels producing NO. After 30 min of the optimal incubation time at room temperature in the dark, the NO generation was maximized due to the complete consumption of the substrates.

The absorbance of the solution was measured after 30 min, using a multimode SpectraMax-M3 microplate reader (Molecular Devices, San Jose, CA, USA) set on UV–Vis absorption mode at the optimum wavelength of 548 nm. A similar measurement was performed with bare PMP fibers (20 knitted threads of 1 × 1 cm^2^ area), taken as a control reference.

According to the standard plot, the absorbance after background correction (subtracting the control absorbance) corresponded to the concentration of released NO from the given volume and the fixed microgel-coated surface area. The resulting NO amount was further divided by the given time and reported as the NO-flux (the released NO per unit area per unit time). Under the defined conditions, the microgel coating provided with the optimized amount of diselenide as a catalytic agent (1.4 mmol%) demonstrates the NO-flux of 7.3 × 10^−10^ mol cm^−2^ min^−1^ before being challenged with blood flow (Figure S1).

The same procedure was conducted for the NO-release assessment from the microgel-coated and uncoated fibers after contact with blood flow, except that the 10 cm long fibers used in the blood experiment were cut into 20 pieces 1 cm long before the Griess assay. The result is discussed in part 3.4, in association with the coating stability.

### 2.5. Blood Donation

For each experiment, 100 mL human blood was withdrawn into two 50 mL syringes, primed with 2.5 IU mL^−1^ of heparin (LEO Pharma A/S, Ballerup, Denmark). The blood was withdrawn from healthy volunteers after informed consent and approval of the ethical committee of the University Hospital of the RWTH Aachen (file no EK134/20). In this matter, blood donations from 10 volunteers (5 male, 5 female) were used for, in total, 10 experiments. Subsequently to the blood donation, each syringe was immediately connected to the syringe pump of the test stand, which was then started simultaneously.

### 2.6. Experimental Groups

Single PMP fibers (OXYPLUS polymethylpentene fibers, 3M/Membrana, St. Paul, MN, USA), part of oxygenators, were used for the evaluation of the microgel coating. For each experiment, fibers were coated for direct comparison against uncoated, bare fibers.

Five experiments were performed without additionally added GSNO. Whereas in five experiments, 2.35 IU mL^−1^ of GSNO was added to the syringes prior to blood withdrawal. Thereby, the influence of externally added substrate for the generation of NO was evaluated.

### 2.7. Test Stand

**Scheme 3:**
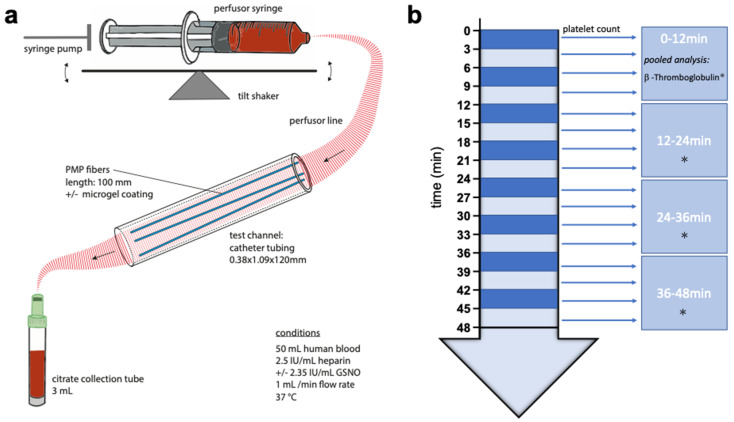
Schematic representation of the test stand (**a**) and blood sampling (**b**). Small arrow: Measurement of platelet count. Star: Pooled measurement of β-Thromboglobulin.

A simplistic presentation of the test stand, mainly composed of a syringe pump, a test channel and their connections, is depicted in Scheme 3a. The syringe pump (SPLG101, KD Scientific Inc., Holliston, MA, USA), holding two syringes of 50 mL blood per experiment, was placed on a tilt shaker, adjusted to 8 cycles per minute, to prevent sedimentation and fractionation of blood components within the syringes. Catheter tubing (PE-Tubing SX02, 0.38 × 1.09 × 120 mm, A. Hartenstein GmbH, Würzburg, Germany) was used as the test channel. For each experiment, three PMP fibers of 100 mm were utilized as test samples and inserted into the test channel. The syringe and test channel were tightly connected via perfusor line (BBRAUN Original Perfusor Line Standard 50 cm, 1.5 × 2.7 mm, B. Braun Melsungen AG, Melsungen, Germany), a cannula (Sterican^®^ G 26, B. Braun Melsungen AG, Melsungen, Germany) and small catheter tubing. The syringe pumps flow was invariably adjusted to 1 mL min^−1^ for each experiment and group.

### 2.8. Blood Sampling and Analysis

Blood sampling was performed continuously while injecting blood through the test channel, as shown in Scheme 3b. Blood, leaving the test channel, after passing the PMP fibers, was constantly collected into citrate collection tubes (S-Monovette, Sarstedt Inc., Nümbrecht, Germany) á 3 mL (one tube every 3 min). A hemogram was measured on an automated cell counter (MEK-6550K, Nihon Khoden Inc., Rosbach, Germany) from pooled blood every 3 min of the experiment. To obtain plasma, the blood samples were centrifuged at 2000 g for 10 min at room temperature and then aliquoted. For subsequent measurements, the single 3-min plasma samples were pooled to quarters (0–12/12–24/24–36/36–48 min) for each experiment and group and stored at −80 °C. Levels of β-TG in the plasma were determined from these quarter samples in accordance with the manufacturer’s instructions by an enzyme immunoassay using β-TG ELISA Kit (Beta- TG ELISA Kit (Human), SEA370Hu, Cloud-Clone Corp., Katy, TX, USA).

### 2.9. Statistical Analysis

A two-way ANOVA with Sidak correction for multiple comparisons and a confidence interval of 95% was performed to verify differences at single time points between the paired groups, using GraphPad Prism software (GraphPad Prism version 9.2.0 for MacOS, GraphPad Software, San Diego, CA, USA, www.graphpad.com, accessed on 18 December 2021). If the calculated *p*-value was <0.05, the results were regarded as significantly different. GraphPad Prism was also used to design the graphs. The presented data are shown as the mean ± SEM.

## 3. Results

### 3.1. Platelet Count and β-Thromboglobulin

**Figure 2:**
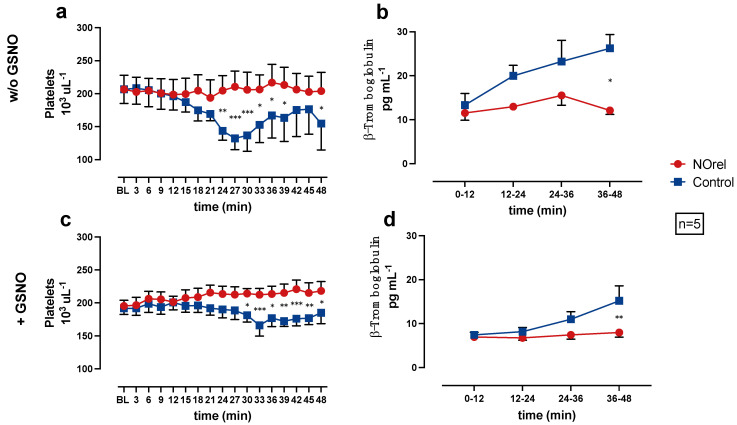
(**a**) Platelets (PLTs) without S-nitrosoglutathione (GSNO)-supplementation, (**b**) β-Thromboglobulin (β-TG) without GSNO-supplementation, (**c**) PLTs with GSNO-supplementation, (**d**) β-TG with GSNO-supplementation over time. * *p* < 0.05. ** *p* < 0.01. *** *p* < 0.001.

To closely evaluate the process of PLT aggregation on the PMP fibers during the experiments, the PLT count (Figure 2a,c) was constantly measured (every 3 min) after passing the test channel.

In the NOrel group, the PLT count remains at baseline levels, while in the Control group, the PLT count decreases significantly compared to the NOrel group. The decrease of PLT count occurs earlier (24 min w/o GSNO vs. 30 min + GSNO) and more intensely w/o GSNO-supplementation (Figure 2a). In the experiments w/o GSNO-supplementation, the PLT count slightly reincreases towards the end of the experiments. The decrease of the PLT count in the Control group can be interpreted as a consequence of PLT aggregation on the bare PMP fibers, whereas the constant PLT count in the NOrel group indicates prevention of PLT adhesion to the fibers.

The β-TG concentration was subsequently measured to the blood-passage through the test channel, in the pooled quarter samples (0–12, 12–24, 24–36, 36–48 min) to examine PLT activation as a result of PLTs contact to the PMP fibers.

Figure 2b,d shows the β-TG concentrations in the pooled samples, each representing a 12-min time interval of the experiment. The Control group shows an increase in the β-TG concentration during the course of the experiments. At the end of the experiments (36–48 min), the increase in the Control group (with and w/o GSNO-supplementation) is significant in comparison to the NOrel group. The β-TG concentration in the NOrel group w/o GSNO (Figure 2b) shows a non-relevant increase and remains at baseline levels with GSNO-supplementation (Figure 2d).

In the series w/o GSNO, β-TG is significantly higher compared to the series with GSNO-supplementation for both groups. The comparison of the experimental series with and w/o GSNO-supplementation shows significant differences for the β-TG concentration in the NOrel and Control group, indicating that the continuous increase of β-TG concentration in the Control group during the course of the experiments is in line with contact activation of the PLTs, whereas the persistent baseline levels of β-TG in the NOrel group indicate no PLT activation. Moreover, it also shows that the addition of GSNO as a supplement is not sufficient to prevent PLT activation in control.

### 3.2. Macroscopic Visual Evaluation

**Figure 3:**
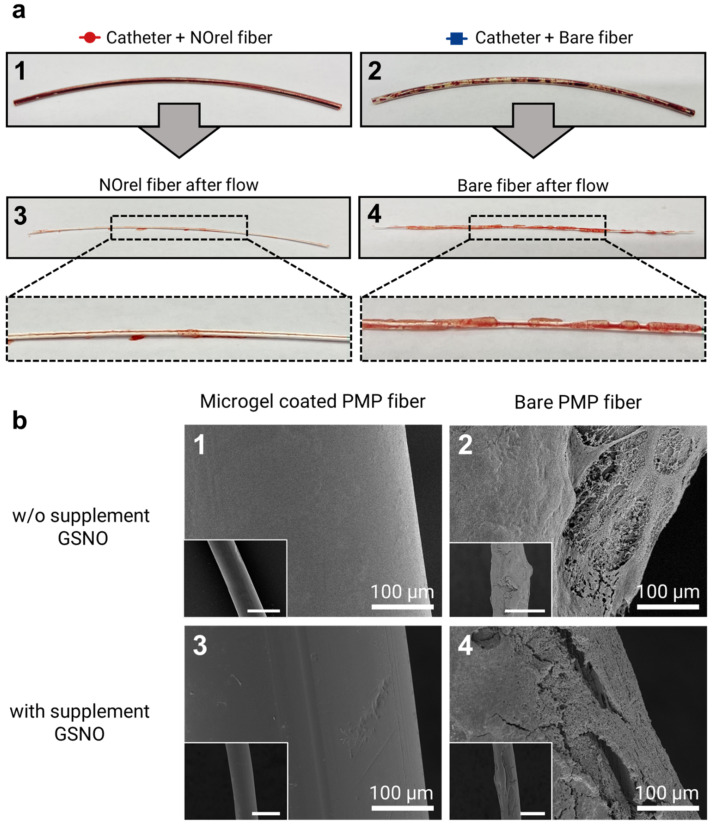
(**a**) Test channel after blood flow experiments for 48 min: the test channel contains (1) coated fibers with NOrel microgels and (2) control bare fibers. Uncoagulated blood can be seen within the test channel (1), while the blood clots are significantly found on the inner wall of the test channel (2). In the same way, the visual inspection of the extracted fibers from the corresponding test channel right after the blood flow experiment shows (3) no stable clot on the NOrel coated fiber, while (4) the significant layers of clot cover the surface of the uncoated fiber. (**b**) SEM analysis of the fibers used in the blood flow experiment: (1) the absence of any fibrin network on the surface of the NOrel coated fiber while (2) a dense layer of clot formed on the bare fiber after contacting the blood flow w/o infusion of supplementary S-nitrosoglutathione (GSNO). (3) A clot-free surface of the coated fiber was observed, while (4) low but still significant fibrin layers settled on the control fiber after exposure to the blood flow with supplementary GSNO. The insets indicate the wide field of view, respectively. Scale bar: 500 µm.

The PMP fibers were inspected visually right after the 48 min exposure to blood flow, followed by an SEM analysis. Visually, NOrel microgel-coated PMP fibers with and w/o GSNO supplementation showed no difference. However, as shown in Figure 3a, there was an evident difference between the coated and bare fibers towards the clot formation. The initial visual inspection of the fibers revealed the presence of uncoagulated blood inside the test channel containing the coated fiber (Figure 3(a1)), while the inner wall of the channel with a bare fiber showed the presence of blood clots (Figure 3(a2)). On extraction of fibers from the channel, the fibers showed similar results; no stable clot was observed on the NOrel-coated fiber (Figure 3(a3)), while the layers of clot covered the contact surface of the uncoated fiber (Figure 3(a4)).

### 3.3. Microscopic Evaluation

To further investigate the effect of microgel coating on clot formation, SEM imaging on coated and bare fibers in both GSNO and w/o GSNO groups was conducted. SEM images of microgel-coated fibers (Figure 3(b1,b3)) show no deposition of the clot, while a dense layer of clot was observed on the bare fiber (Figure 3(b2,b4)). Although the addition of GSNO to coated fibers did not show any significant difference in clot formation, the presence of additional GSNO moderately reduced the amount of clot formation on the bare fibers. It is well established that naturally occurring concentrations of GSH in blood have the potential to reduce GSNO to release NO; however, the extent of NO released by this mechanism is quite low [42].

These promising results of the microgel coating in the absence of any supplementary GSNO infusion showed that the microgel coating is catalytically sufficient to endogenously release NO from the bioavailable sources in blood.

### 3.4. Coating Stability

The measurement of NO-flux after blood flow indicates the surface release of 5.9 × 10^−10^ mol cm^−2^ min^−1^ (Figure S1), which affirms the stability, as well as the maintained functionality of the coating under flow conditions. The drop in the NO-flux from 7.3 × 10^−10^ mol cm^−2^ min^−1^ after blood flow was expected due to the possible reduction of diselenide bonds and the formation of new selenenyl sulfide (Se-S) bonds after long-term contact with the reducing environment of blood. However, the result is still remarkable since, despite the slight drop, the coating maintains the NO-flux in the range of an in vivo flux [26].

## 4. Discussion

This study investigated the antifouling and anticoagulative properties of PMP fibers coated with NOrel microgels under flow conditions. It gives an insightful view into the local PLT-inhibitory potential of the microgel coating in dealing with a dynamic flow and accompanying shear stress, which increases protein deposition and PLT adhesion [34]. For this purpose, microgel-coated PMP fibers were inserted into a test channel and exposed to a continuous flow of heparinized (2.5 IU mL^−1^) human blood for 48 min. For a direct comparison, bare PMP fibers were examined simultaneously and in the same way. The combined analysis of the fibers macroscopic and microscopic (SEM) visualization and the measurement of PLT count and activation (β-TG) showed clot formation on the bare PMP fibers are consistent with a significantly decreased PLT count and increased PLT activation in comparison to the microgel-coated fibers.

### 4.1. NOrel Microgel Coating

The immobilization of microgels on the highly hydrophobic PMP fibers was facilitated by a surface-adhesive anchor peptide called liquid chromatography peak I (LCI), bearing a strong binding affinity to biologically inert polymers [35]. The amphiphilic structure of LCI enables adaptive interactions of the peptide upon exposure to a surface through a conformational transformation. The recombinant LCI was produced as a fusion protein with a functional block consisting of an enhanced green fluorescent protein (eGFP), bearing the free cysteines fused to the N-terminus of LCI [43]. This facilitated the fluorescent detection of the anchor peptide on the eGFP-LCI-treated surface (Scheme 3a) and covalent attachment of the microgels through the interaction between the eGFP-thiols and the microgel-oxirane moieties introduced by GMA to the microgel structure (Scheme 3b) [29].

### 4.2. Mechanism of Endogenous NO-Release

Natural endothelium continuously releases endogenous nitric oxide (NO) to inhibit PLT activation [26,44] with a surface flux of 0.5–4 × 10^−10^ mol cm^−2^ min^−1^ [6]. The released NO activates the generation of cyclic guanosine monophosphate (cGMP) and induces a variety of further-downstream effects leading to the reduction of αII_b_b_3_ integrin activity and eventually the inhibition of PLT activation and aggregation [45]. NO release can be facilitated from S-nitrosoproteins (e.g., S-nitrosoalbumin (SNO-Alb) and S-nitrosoglutathione (GSNO)) by an enzyme, Glutathione peroxidase (GPx). This process of NO release from GSNOs is mediated by the diselenide catalytic center of the enzyme. Due to the reducing nature of the diselenide bonds, it can reduce the S-nitroso bond (S–NO) of S-nitrosoproteins present in the blood to release NO [46].

To mimic this function in our coating, microgels with diselenide crosslinks were introduced. The essential steps of this catalytic mechanism involve the bioavailable concentration of GSH reducing agent present in the blood, which reversibly reduces the diselenide compound to the primary active species of selenol/selenolate (RSeH/RSe¯). The obtained active species mimics the GPx-like enzymatic function and facilitates the decomposition of the endogenous NO-donors leading to NO-release. To maintain the catalytic cycle, GSH with the generated selenenyl sulfide (RSe-SG) intermediate reproduces the active species of selenol/selenolate [29,46,47,48] (Figure S3).

Since the bioavailability of S-nitrosoproteins in vivo is constantly replenished, such an endogenous release mechanism promises long-term maintenance of physiological NO flux at the blood-contacting foreign surfaces interface.

Our result demonstrates that even in the flow condition, the NO flux generated from the microgel coating was sufficient in the prevention of PLT activation. The results show that bioavailable GSNO is sufficient for sustaining the physiological NO flux. This is a landmark result, as this will allow us to upscale the system and calculate the amount of diselenide crosslinks required per cm^2^ of the microgel-coated PMP fiber for a given volume of blood for future in vivo studies.

### 4.3. Platelet Aggregation and Activation

If blood comes into contact with foreign surfaces, such as the test channel and the fibers in our setting, adhesive plasma proteins, such as fibrinogen or von Willebrand factor, adhere to the bare PMP fibers [49]. Subsequently, PLTs get activated by the contact of the GPIIb/IIIa receptor to fibrinogen, connect and aggregate to the PMP fibers surface [50]. Due to this PLT aggregation, the measured PLT count behind the test channel decreases, as PLTs aggregated on the PMP fibers are not counted.

The delayed and moderate PLT decrease in the Control group with GSNO-supplementation can be explained by the effect of the GSNO. GSNO is a substrate for NO generation and can serve as a NO donor by the decomposition or metabolism even without microgel coating on-site [51]. This NO release due to sole GSNO-supplementation could reduce PLT aggregation on the bare PMP fibers. The proceeding PLT aggregation on the bare PMP fibers throughout the experiment is presumed to diminish the available free space on the surface of the fiber for PLTs to adhere. This could explain the slight reincrease of the PLT count in the Control group without GSNO.

β-TG is a reliable marker of PLT activation, as it is released from the α-Granules of activated PLTs [15]. Thus, the continuous increase of β-TG concentration in the Control group during the course of the experiments indicates PLT activation due to PLT and protein interaction with the bare PMP fibers, whereas the persistent baseline levels of β-TG in the NOrel group indicates no PLT activation. These measurements confirm the findings of thrombus formation on the bare fibers, respectively, the clean, coated fibers that were protected by the NOrel microgel coating, inhibiting PLT activation.

### 4.4. Test Setting

The test setting, especially the output of flow via syringe pump, the size of the test channel and amount of anticoagulation (2.5 IE mL^−1^ heparin), has proven successful in providing conditions to sustain constant blood flow over 48 min but simultaneously allowing clot formation on the PMP fibers surface.

Nevertheless, the test stand is limited, as it represents a miniaturized in vitro test setting, lacking organs and cells, as well as adequate size and volumes in comparison to clinical ECMO. Therefore, this study has limited validity for in vivo translation or application. Another shortcoming is the absence of circulation; the test setting adequately generates blood flow, but no recirculation. A test circuit with constant recirculation of blood [52,53] is preferable but is not yet feasible for miniaturization, especially due to missing mini-centrifugal pumps, providing less shear forces in a test circuit in comparison to peristaltic roller pumps.

## 5. Conclusions

In this work, we show that the integration of hydrophilicity and NO-release functions of the microgel coating could prevent clot formation in the flow condition, with minimally heparinized blood. The highly hydrophilic surface of the microgel coating removes the protein fouling, and the incorporated diselenide functionality of microgels facilitates the endogenous release of NO from the blood pool of NO-reservoirs to inhibit the PLT activation and thereby prevents a stable clot formation on the surface of the oxygenator membrane fibers, in contrast to bare fibers. The endogenous release of NO from the biomimetic microgel coating does not depend on the finite reservoir of NO donors; rather it depends on the endogenous source of NO in the blood, which is constantly replenished in vivo; this shows the high potential of such coatings to be used for long term and sustained NO release. The PLT inhibitory effect of the NOrel-coated PMP fibers is localized to the proximity of the coated fibers, which guarantees no systemic NO (half-life in blood 5 s and diffusion 160 µm) side effects, such as vasodilatation, and will assist in maintaining the hemostasis of NO in the blood.

As an outlook, the in vivo evaluation of the microgel coating in general, e.g., in animal trials [54,55], as well as the in vitro evaluation in a test-circuit for an extended period of time, would be desirable and insightful.

## Data Availability

The datasets generated and/or analyzed during the current study are available from the corresponding author upon reasonable request.

## Supplementary Materials

The following are available online at https://www.mdpi.com/article/10.3390/membranes12010073/s1, Figure S1: NO-flux profile of coating before and after blood flow, Figure S2: The proposed mechanism of NO-release, Figure S3: High-resolution N 1s XPS spectra of control and coated fibers before and after HBS, Table S1: The elemental composition of coated and control fibers before and after HBS.
