# Supplementary material for: Endogenous Nitric Oxide-Releasing Microgel Coating Prevents Clot Formation on Oxygenator Fibers Exposed to In Vitro Blood Flow
Source: Membranes (Basel). 2022 Jan 6;12(1):73. doi: 10.3390/membranes12010073 (PMC8779597; doi:10.3390/membranes12010073)
Supplement: Supplementary file 1 [file membranes-12-00073-s001.zip › membranes-1504661-supplementary.pdf]

membranes

Supplementary Information

Endogenous nitric oxide-releasing microgel coating prevents clot formation on oxygenator fibers exposed to in vitro blood flow

Patrick Winnersbach [1,#], Aisa Hosseinnejad [2,#], Thomas Breuer [3], Tamara Fechter [1], Felix Jakob [2], Ulrich Schwaneberg [2,4] , Rolf Rossaint [1], Christian Bleilevens [1,*] and Smriti Singh [2,5,*]

1 Department of Anesthesiology, University Hospital RWTH Aachen, Pauwelsstraße 30, 52074 Aachen, Germany; pwinnersbach@ukaachen.de (P.W.); tfechter@ukaachen.de (T.F.); rrossaint@ukaachen.de (R.R.); cbleilevens@ukaachen.de (C.B.)
2 DWI—Leibniz-Institute for Interactive Materials e.V., Forckenbeckstr. 50, 52056 Aachen, Germany; hosseinnejad@dwi.rwth-aachen.de (A.H.); jakob@dwi.rwth-aachen.de (F.J.); singh@dwi.rwth-aachen.de (S.S)
3 Department of Intensive Care and Intermediate Care, University Hospital RWTH Aachen, Pauwelsstraße 30, 52074 Aachen, Germany; tbreuer@ukaachen.de
4 Institute for Biotechnology, RWTH Aachen University, Worringerweg 3, 52074 Aachen, Germany; u.schwaneberg@biotec.rwth-aachen.de (U.S)
5 Max-Planck-Institut für medizinische Forschung, Jahnstraße 29, 69120 Heidelberg, Germany
* Correspondence: cbleilevens@ukaachen.de (C.B.); smriti.singh@mr.mpg.de (S.S)
\# These authors contributed equally to this work.

Citation: Winnersbach, P.; Hosseinnejad, A.; Breuer, T.; Fechter, T.; Jakob, F.; Schwaneberg, U.; Rossaint, R.; Bleilevens, C.; Singh, S. Endogenous Nitric Oxide-Releasing Microgel Coating Prevents Clot Formation on Oxygenator Fibers Exposed to In Vitro Blood Flow. *Membranes* **2022**, *12*, 73. https://doi.org/10.3390/membranes12010073

Academic Editor: Bettina Wiegmann

Received: 26 November 2021
Accepted: 28 December 2021
Published: 6 January 2022

Publisher's Note: MDPI stays neutral with regard to jurisdictional claims in published maps and institutional affiliations.

Copyright: © 2022 by the authors. Licensee MDPI, Basel, Switzerland. This article is an open access article distributed under the terms and conditions of the Creative Commons Attribution (CC BY) license (https://creativecommons.org/licenses/by/4.0/).

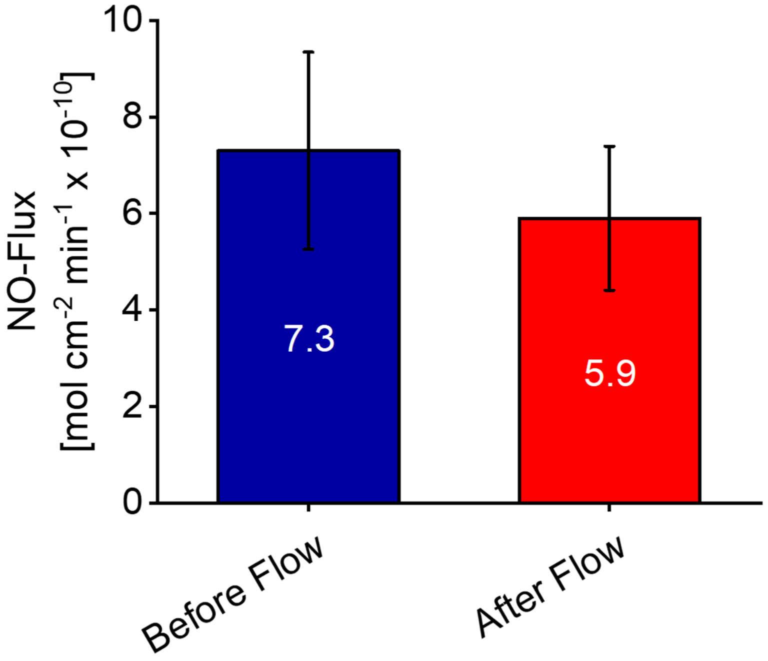

Figure S1. NO-flux profile from 1 × 1 cm² coated PMP fiber surface in the presence of the bioavailable concentration of L-Glutathione (GSH) (1 mM) and S-nitrosoglutathione (GSNO)(7 µM) before and after blood flow measured by Griess test at 30 min (n = 2–3).

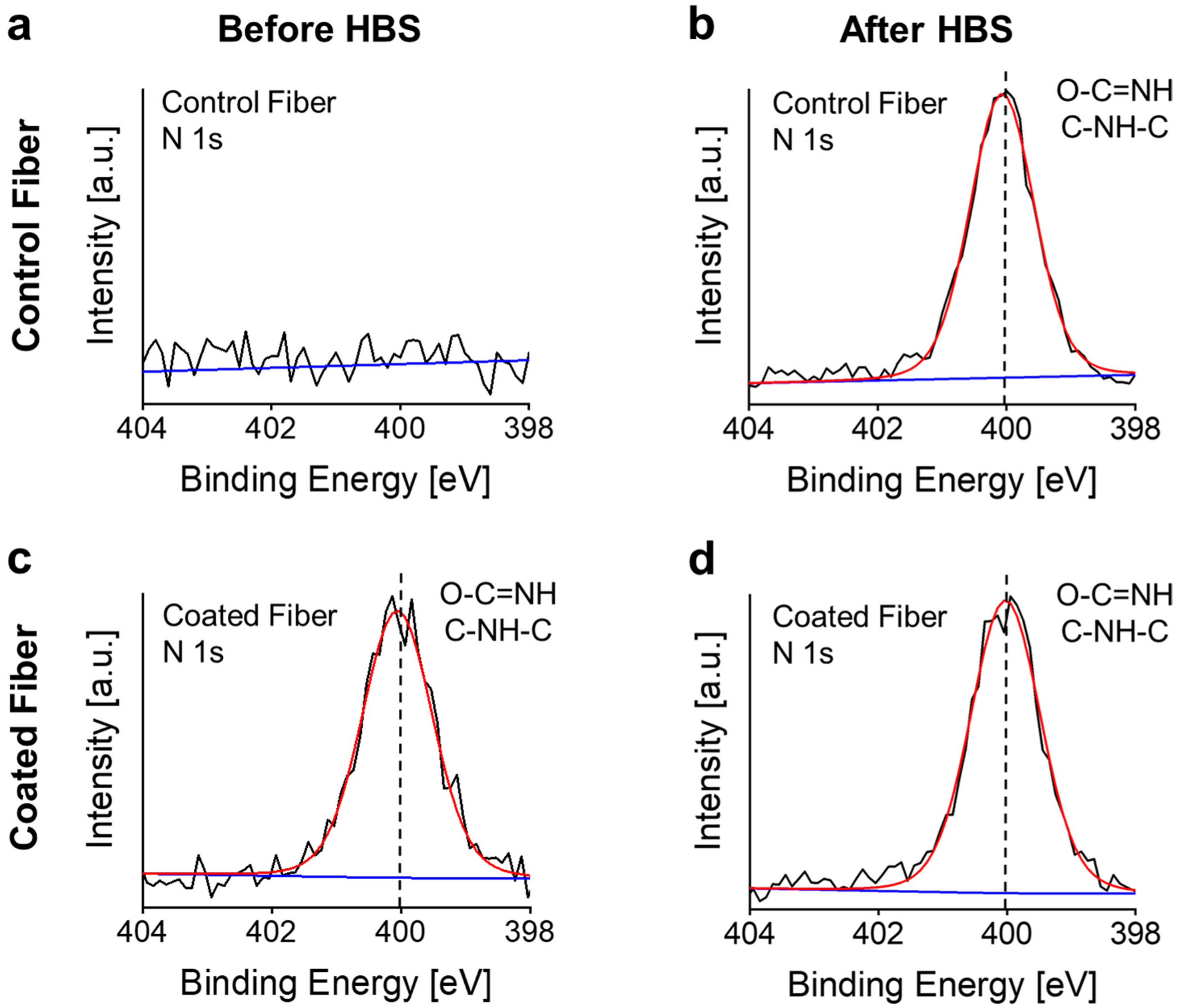

Figure S2. High resolution N 1s X-ray Photoelectron Spectroscopy (XPS) spectra of the control and coated fiber (**a**,**c**) before and (**b**,**d**) after exposure to human blood serum (HBS).

$$\mathrm{RSe{-}SeR + GSH \rightleftharpoons RSe{-}SG + RSe^{-} + H^{+}} \quad (1)$$

$$\mathrm{RSe{-}SG + GSH \rightleftharpoons RSe^{-} + H^{+} + GS{-}SG} \quad (2)$$

$$\mathrm{RSe^{-} + H^{+} + R'S{-}NO \rightleftharpoons \tfrac{1}{2}\,RSe{-}SeR + R'SH + NO} \quad (3)$$

$$\mathrm{RSe{-}SeR + 2\,R'S{-}NO \rightleftharpoons 2\,RSe{-}SR' + 2\,NO} \quad (4)$$

Figure S3. The proposed mechanism of NO-release from S-nitroso compound (R'SNO) by organoselenium catalyst (RSe–SeR) mediated by a reducing agent (L-Glutathione (GSH)). The other indicative species are selenosulfide (RSe–SR' and RSe–SG) and the conjugate base of selenol (RSe^{-}).

Table S1. The elemental composition of the coated and control fibers before and after HBS.

	**Coated Fiber**			**Control Fiber**		
Before HBS	C<br>62.0%	N<br>7.2%	O<br>30.8%	C<br>81.6%	N<br>—	O<br>18.4%
After HBS	C<br>56.0%	N<br>7.5%	O<br>36.5%	C<br>57.7%	N<br>9.7%	O<br>32.6%
